# Annual Session of American Dental Convention, Southern Dental Association and Maryland and District of Columbia Dental Society, in Joint Session

**Published:** 1877-10

**Authors:** J. B. Hodgkin


### AMERICAN JOURNAL

OF

### DENTAL SCIENCE.

**Vol. XI. THIRD SERIES—OCTOBER, 1877. No. 6.**

### ARTICLE I.

### *Annual Session of the American Dental Convention, the Southern Dental Association, and the Maryland and District of Columbia Dental Society, in Joint Session.*

Reported for this Journal by Prof. J. B. Hodgkin.

FIRST DAY'S PROCEEDINGS.—Morning Session.

The annual session of the American Dental Convention, the Southern Dental Association and the Maryland and District of Columbia Dental Association commenced at Oakland, Md , in Memorial church, Tuesday, August 14th.

Dr. W. H. Atkinson, of New York, President of the American Dental Convention, presided over the opening exercises, with Dr. A. C. Ford, of Atlanta, Ga., Vice President of the Southern Dental Association, and Dr. R. F. Hunt, of Washington, D. C., President of the Maryland and District of Columbia Dental Society, on the platform with him.

An address of welcome from the Maryland and District of Columbia association to those assembled was read by Dr. Nelson, of Frederick, Md., reviewing briefly the history of dentistry in the past, its present status, and what may be expected of it in the future. At the conclusion of his address, Dr. Atkinson, in the absence of any prepared address made a few remarks explanatory of the origin of the American Dental Convention, its rise and progress. The learned and unlearned had participated in its organization, the latter, as is always the case in such organizations, being the most strenuous in laying down stringent rules by which others are admitted, and so in consequence of the difficulties incident to its organization, there arose in 1860 another society, the American Dental Association.

An earnest desire had always existed to merge into one body these separate and antagonistic elements, and his acceptance of the position of president last year was with a hope that such a reconciliation might be brought about; he had an earnest desire to heal this schism, but efforts in the past had been failures, at which it perhaps is as well to rejoice— certainly no great cause for regret. No committee from the American Dental Association had met the advances of the American Dental Convention, and so the proffers of reconciliation on the part of the convention were a failure. Still, at the last meeting of the former body a committee was appointed, which will doubtless shape a report which will command respect, and possibly conciliate the two bodies. It would be desirable if all these societies could be so constructed as to render it no longer desirable that the American Dental Convention should exist.

Whatever else we may do, let us here throw aside all envy and bickering, and with a sincere desire to learn the truth, give our hearts to this work; so acting as to give the lie to the terrible suspicion which obtains among the public that we come from these gatherings more fully prepared to wrench unclean money from our patients.

At the conclusion of his remarks, Dr. Ford, of Atlanta,

Ga., Vice-President of the Southern Dental Association, in the absence of Dr. Cobb, the President, made a few remarks, explaining the status of the body he represented, as to its non-sectional character, its aims, &c., expressing himself as a worker rather than a talker, and rejoicing at the fraternal feeling which made it desirable that three such societies should thus unite, feeling sure that good would ensue.

Dr. R. F. Hunt, of the Maryland and District of Columbia society then read his annual address. Adverting to the fact that there were here assembled three distinct societies, it marked a large advance in union of sentiment, and showed the onward march of dental surgery, and with this advance our responsibility has increased. We have reached a point where our investigations must no longer be partial, but must embrace all the organism, and our researches carry us back among past generations.

The profession is equal to the demand made upon it, and its new phases are met and grappled with in true scientific spirit. It would be well, however, for us to ask ourselves, each man of us, what are we doing for dentistry? Are we earnestly endeavoring to add our strength to the common fund, or are we content that others shall work for us?

He thanked the Maryland and District of Columbia Association for the honor of the post he held, welcomed Drs. Atkinson and Ford and the associations they represented and presided over, and concluded with an earnest hope that all jealousies and bitter feelings would be laid aside, and all join in one united effort for the common good of the human race.

Dr. Atkinson, Dr. Hunt having concluded, rose and stated that this was a joint meeting of three associations, and they had so prepared the programme as to hold the sessions together, in such a way as not to commit any person present to the support of more than one body.

Dr. Waters read the minutes of last year.

Dr. Atkinson: As there is no quorum of the American

Dental Convention present, action on the minutes will be laid over.

The reading of the minutes of the Maryland and District of Columbia Association was dispensed with, their printing ordered, and on motion of Dr. Coy, the meeting adjourned to meet at half-past three.

### Afternoon Session.

Dr. Atkinson, in calling the convention to order, stated the terms of admission to the American Dental Convention, and also to the other associations. In seeking for a term by which to style this meeting he would perhaps call it a "mass-meeting," as it partook more of this character than of a regular organization.

Prof. J. B. Hodgkin, of the Baltimore College of Dental Surgery, and Dr. B. M. Wilkerson, Demonstrator of Operative Dentistry in that institution, and Dr. E. M. McSherry, of Frederick, Md., were elected active members, and S. Waterman, M. D., of New York, T. B. Evans, M. D., of Baltimore, elected honorary members.

Dr. S. Waterman had, it was stated, an interesting lecture on the Spectroscope and arrangements were made by which, at an early day, the Associations could have the benefit of hearing it.

A number of committees were called, and no reports being made, the subjects were passed for the time.

Dr. Ford read a paper written by Dr. E. Parsons, of Savannah, Ga., on Dynamic or Vital Forces, and at its conclusion it was discussed by Dr. Atkinson, who said that one statement of the paper was well worthy of thought and that was, that we were better prepared to do than to understand what we do.

Reports from a large number of committees were called for, without eliciting responses; some one stated that none were at hand.

A letter was read from Dr. Fouke, of Maryland, stating that in lieu of a paper on Mechanical Dentistry, promised ∡q him, he would send one on Æsthetical Art. Adjourned

### SECOND DAY'S PROCEEDINGS.—Afternoon Session.

The Joint Convention assembled at half-past three, Dr. Atkinson in the chair.

Dr. C. Palmer, of New York, took the floor on the subject of Operative Dentistry, illustrating his remarks by reference to a number of diagrams of the front teeth, showing the damaged condition of the teeth from mechanical abrasion, and his manner of filling such teeth, restoring the lost tooth-form, &c. He prefaced his address with the sentiment that all his efforts were based on an earnest desire to help and benefit his fellow-men, and to that end he made his teaching as much as possible by demonstrations. He got his impressions from unknown sources, and could only work as he was helped.

Our tendencies are in these meetings toward the higher developments and phases of scientific discussion, and to the avoidance of details. It was to these last that he hoped by directing attention to do some good.

His subject, the filling of teeth, involved many preliminaries which he would pass over and take up the steps involved in—

1st. The adjustment of the rubber-dam.

2nd. Separation of the teeth.

3rd. Preparation of the cavity.

This last step in this process is made, always keeping in view the design of using only the most cohesive gold—he uses no other—and hand malleting, using serrated points.

To deal with difficult cases was desirable, as most of us only needed instruction here, plain cases being within the mastery of all.

The first step, the adjustment of the rubber dam, was not difficult with properly constructed clamps; some of the best were invented by his son, Dr. Delos Palmer, and indeed he was the originator of the rubber-dam clamp. His plan for constructing a clamp was to take an impression of the tooth—he referred to third molars, posterior and buccal cavities being those under consideration—make a plaster model, fit a sheet—lead pattern on this, shaping it as desired and copy this in sheet steel, fitting this with great care and tempering.

A special feature of these clamps is the lowering of the bow of the clamp, thus clearing the adjacent parts and getting it out of the way of the operator.

If he adjusted the rubber-dam without a clamp he would commence at the tooth anterior to the one he wished to operate on ; if he used a clamp on the other hand he commenced at the back and worked forward.

As to the thickness of the rubber-dam, he used only the thin—the thick rubber was cruel to the patient and unmanageable.

The cavity was so shaped as to provide for a cohesive gold filling. He carried the gold towards the walls, filling around the side first. He was satisfied that cohesive gold made the tightest stopping. His method differed somewhat from that pursued by some operators ; he used the gold in large pieces, and in loose folds. He also cuts retaining points straight, not undercut, and such points as he may drill are drilled very small. He used retaining screws only occasionally, and preferred not to depend on them. The gold itself was No. 4, not annealed by the maker—he was sure that this was useless and injurious, each heating doing harm. He heated it to redness, and even to whiteness. He also illustrated a simple method of rolling gold into rope by the use of towel He reiterated that the gold must come from the maker beaten, not annealed at all. Such gold could be kept indefinitely, and made as cohesive as desired when wanted. E. Kearsing, 104 Hoyt street, Brooklyn, N. Y., made the only gold he could use, making it after his special directions.

At the conclusion of his remarks the meeting adjourned to meet at half-past seven at night, to resume discussion of operative dentistry.

NIGHT SESSION.

Meeting assembled at half past seven, when Dr. Walker, of New Orleans, read a paper written by Dr. McLane, on pathological conditions of the pulp, giving a history of the nature and condition of exposure of the tooth pulp, the method of reducing congestion, inflammation, &c., and giving the therapeutic and surgical treatment of such cases.

Dr. Coy then read the report of the committee on operative dentistry, which, covering many points, was too brief in each to admit of an abstract being made. It claimed for most operations greater success if performed with soft foil, using cohesive, as an exception. The record of soft foil was no uncertain one on this subject, too much lasting and durable work having been done by old operators to render the question of the usefulness of soft foil unsettled.

At the conclusion of the reading of the report, Prof. J. Taft, of Cincinnati, Ohio, discussed the first paper read, that on the pulp, advocating strongly the use of all possible means for the preservation of the pulps of teeth, and claiming that no one did his duty to his patient who acted otherwise. He hoped the day was at hand when all pulps would be saved alive. He was fully assured in his own mind thatthe pulp was no useless piece of tissue which had served its purpose in the development of the tooth, and could be destroyed with impunity, but that it was the organ by which the tooth was nourished, and in case of its death much more speedy breaking down of the dentine occurred. Dead teeth were also much more liable to abscess and pericemental disease. He discussed the various conditions of the pulp exposure from decay, wear, and the accidental exposure by an operation, and on these depended the treatment. Accidental exposure he thought could always be capped at once, while that arising from decay would usually require treatment, especially if disease was, as is usually the case, present. The capping material should possess certain properties to render it useful; it should be such as would not irritate the pulp, be easily adjusted with the most perfect exactness against the pulp without pressure, and should be durable in itself. It should be acceptable to the pulp chemically, should not be rough, should be a non-conductor of thermal changes. A majority of his cases of capping he thought were successes.

---

THIRD DAY'S PROCEEDINGS.—Morning Session.

The convention assembled at 11 A. M.

Dr. C. E. Busey, of Baltimore, being proposed for membership, Dr. E. P. Keech moved to ballot for election. This motion was lost and Dr. Busey was elected, *viva voce*, a member of the Maryland and District of Columbia Dental Society.

A paper on anæsthesia by Dr. H. C. Thompson, of Washington, D. C., was read. The line of investigation pursued by the committee proposing the paper has two objects in view: 1st. To substantiate the investigation of others, and 2nd, To pursue an original line of research, making this as new as possible. Anæsthesia was certainly the most dangerous condition of life, and the most daring should hesitate to induce it. He considered the essentials of this condition to be a diminution, oxygenation, and a retardation of assimilation; and its three stages were perhaps only differences in degrees of action. He disputed the propriety of the usual preparatory preparation of the patient for anæsthesia by the use of diffusible stimulants as wrong in principle, as the patient should approach and pass into this state, in as nearly a normal condition as possible. He considered that chloroform had direct power to arrest the action of the heart

As to restoratives, reflecting that the anæmic condition of the brain was probably secondary to the cessation of the action of the heart, he thought Nelaton's famous method of inverting the position of the patient, *i. e.*—standing him on his head, with a view of causing a return of blood to the head, as less efficient than electricity, inasmuch as this latter could be made to act directly on the heart itself, the organ at fault. The writer referred to the frequent impurities of chloroform as one of its dangers, and showed the methods of detecting them. The paper was in all respects one of careful preparation, and to which a brief abstract does injustice.

S. Waterman, M. D., of New York, then took the floor, and occupied the most of the morning in an address partly written and partly extempore on Spectroscopic Analysis, including a brief history of that instrument and its uses. In introducing the subject of spectroscopic analysis, he assumed that as all anæsthetics were more or less dangerous, it was of vast importance that their methods of action on the blood should be fully known beforehand, that the administrator might be fully aware of the condition he is bringing about. The physical signs of danger from anæsthesia are vague and contradictory, while the chemical results are certain, accurate and easily seen, and it is to these last that he would call the attention of his audience as best determining the action of anæsthetics. As the chemical constitution of anæsthetics is so different, it is an error to suppose, as some seem to do, that they are alike in their action; and the only accurate knowledge of the difference in their modes of action is found through the Spectroscope. He spoke of the Hydrate of Amyl, and of Dr. Richardson, a most thorough investigator on this subject, this being a substance possessing apparently the curious property of annulling consciousness of pain, but leaving active a knowledge of what was going on; the agent itself however having proven dangerous.

Giving a brief history of the spectroscope, and exhibiting an instrument he had with him, he proceeded to show by diagrams and verbal description the functions of this instrument in decomposing light into spectral bands of different colored rays, as familiarly seen in the rainbow, with the addition of many minute sub-divisions and lines, known from their discoverer as the lines of Fraunhoffer, and first described by him in 1814. A marked difference in the constitution of solar and artificial light was that in the solar spectrum, these lines or bands as they are usually called, are interrupted, whilst in the latter they are plain. These solar lines are capable of being photographed, and it was a remarkable proof of the accuracy of different observers that a photographed copy of a spectrum made in this country was precisely like one copied by hand in Europe, showing that the spectrum was the same under all circumstances.

This instrument was of immense interest to the scientific world, as demonstrating the constitution of remote bodies. Any substance capable of combustion on being burned, and its light decomposed into the spectrum, developed certain lines. Sodium was characteristic, so was hydrogen, and so indeed every substance known to chemistry; all gave distinct and constant results. So in this way the light of the sun was demonstrated, to be composed largely of hydrogen, and even the fixed stars and nebulæ were analyzed and their constitution made known. It was worthy of notice, that in these latter no bands, but only spots were seen, probably proving that no solid matter existed. A beautiful illustration of the benefits conferred on the arts by science, was shown in the use of the spectroscope in the manufacture of Bessemer Steel. This instrument was made use of to determine the exact amount of decarbonization of the cast iron, and so the quality of the steel determined with an accuracy, altogether impossible or even approachable in any other way. The whole art of steel manufacture had thus been revolutionised, simplified and made exact.

Understanding that no two sample substances during combustion give the same bands in the spectroscope, and substances of a complex sort, and whose chemical character would be destroyed in the combustion, as liquids, may be examined by passing light through them, and the least change in their chemical constitution detected, we are prepared to understand the value of the spectroscope in demonstrating any changes, which may take place in the blood during anæsthesia. As an illustration of how infinitely small an atom of any substance may be detected, it is shown that the presense of the one three-millionth part of a grain of sodium may be instantly shown by this instrument. And a remarkable instance of its exactness is seen in the history of the chemist Bunsen, who, on examining the water of a mineral spring in Germany, detected a a new spectroscopic line, and consequently a new metal, and evaporating forty tons of the water, obtained two hundred grains of a metal heretofore unknown.

It is characterstic of human blood in a healthy condition, that two certain and constant bands are seen in it by spectroscopic analysis; these indicate oxygenated blood, and may be removed by withdrawing the oxygen with an air pump, and restored by shaking it up again with ordinary air. Blood which is old can be thus restored. The removal of oxygen is always accompanied by the substitution of a black band, for the two dark ones that were before seen. Now in the treatment of the blood with nitrous oxide gas it is seen that a permanent decomposition of the blood takes place—it can no longer be restored—its composition is altered; when once the blood is saturated with this substance it can no longer be re-oxygenised, the injury is permanent. The exactness of these tests lead to conclusions that are unavoidable, *i. e.* that nitrous oxide gas is the most dangerous, instead of the most harmless of anæsthetics, and that in the use of no agent are there so many hair-breadth escapes from death.

Nitrous oxide is not a true anæsthetic. It acts by suspension of one of the most important functions, that of respiration. And its remote effects in producing disease are so common, as to be noticed by all. Numerous cases are on record, of nervous and other diseases being produced, and of death resulting from its use.

He adverted briefly also, to the dangers attending the use of chloroform, showing how it acted by dissolving certain crystalline substances in the blood. Its high boiling point was a grave objection to its use; but the gravest objection of all was found in that its tendency was to produce sugar in the urine, which substance he had repeatedly detected after its administration. The best restoration he thought for narcotism from chloroform was the inhalation of air, heated to 130° F., this being the boiling point of chloroform.

Ether acts quite differently, dissolving certain substances out of the blood, and forming a gelatinous mass.

At the conclusion of the address the meeting adjourned till the afternoon.

### Afternoon Session.

Dr. R. B. Winder, of Baltimore, stated that the Baltimore and Ohio Rail Road Company had kindly offered to give the associations an excursion to the Cheat River, and it was for the association to accept the offer and fix the time. This was done, and Sunday morning was agreed on as the time for the excursion.

Dr. Caldwell, of Baltimore, took the floor on the consideration of the address of Dr. Waterman.

The origin of the spectroscope was due to that genius in chemistry, Michael Faraday, who when a boy, a student of Sir Humphry Davy, had first observed the phenomena which since has taken so important a place in chemistry, and which we have seen may play so important a part in the subject under discussion.

The first universal spectroscope was the flowers which, absorbing certain rays and reflecting others, gave us the colors we find so pleasing to the eye. This is the shadow world where light and shade give results wonderful, beautiful and complex. He thought that as we could read in the flowers the chemical constitution of light and the play of forces, so, if it could be rightly read, the human face might be found a spectroscope where motives and thoughts could be analyzed and read as in a glass. He had hoped that some demonstration and vivisections could have been made here to illustrate these beautiful ideas of Dr. Watermar, but it was found impracticable. Adjourned.

### Night Session.

Dr. Caldwell resumed his remarks on the paper of Dr. Waterman, on the dark lines of spectroscope exhibited when blood was subjected to the action of Nitrous Oxide Gas. He spoke of the dark line called by Dr. Waterman, the "death line," and dwelt on the curious condition sometimes present in anæsthesia, when the consciousness of pain was in abeyance, while ordinary consciousness was in active operation. As the nerval tracts, over which the nerve current passes in both directions, is the same, it was difficult to understand how this could be.

Dr. E. P. Keech asked if Dr. Waterman could distinguish by the spectroscope old blood, *i. e.*, blood drawn from the body and kept a long time from blood in abnormal condition freshly drawn. Dr. Waterman in reply said this was a question he had never investigated. In reply to a question of Dr. Caldwell, he said, that the results of science are exact, and as a scientist he was bound to accept them, however they might clash with preconceived theories or common observations. If the results of scientific observations did not coincide with what seemed to be the previously known facts of the case he could not help it. As a candid observer he must accept what science demonstrated as the truth. If these hard facts which science develops are hard he could not help it. Brown-Sequard had demonstrated that the removal of the supra renal capsules covered the crystalization in the blood of a substance previously in solution, the hæmato-crystalline. He claimed that in anæsthesia by nitrous oxide, certain permanent changes in the blood were effected, which even electricity could not break up,—the blood was permanently disorganized. The debate on the subject here took a conversational shape, in which the question as to the power of hæmato-crystalline to dissolve carbonic acid in the blood, was discussed; the statement made that the blue color of venous blood was due to the carbonic acid in it, and the re-demonstration by Dr. Waterman that the dark band in putrid blood, and the dark band in blood which has been subject to the action of nitrous oxide gas are invariably the same; and that so far as these demonstrations go, the blood subject to the action of this gas, is permanently and irrecoverably disorganized.

Dr. Caldwell spoke of the uses of electricity in dissipating the tumors, and also in diagnosing their character. He illustrated this by quoting some examples, in which ovarian tumors were diagnosed by the electric current. He had also made a fluid extract of beef, by the decomposition of beef in the electric current.

Dr. Waterman, in answer to a question on the subject of the transfusion of blood said, that he was of opinion that all blood was not fit for transfusion, even that of the same species, and that many of the failures in this direction were due to a want of fitness in the blood used for this purpose. There was a decided difference in the specific gravity of different samples of blood, and this was a point of importance. He had at one time thought that the hæmato-crystalline in the blood could be used as a medicine in those cases where changes had been wrought in the blood, but so many difficulties were in the way that the idea had been abandoned; the chief danger being that some septic change might occur in this substance by long keeping.

Dr. Atkinson expressed himself as always having been opposed to any agent that destroyed action and consciousness; and thought all anæsthetics were of the devil. If the heart beat be fully stopped he had supposed that the subject died infallibly.

Dr. Waterman, who seemed to have been misunderstood, said that if the hæmato-crystalline be destroyed in the blood cells the patient died; this was true, and it was true as he had demonstrated that nitrous oxide destroyed this substance, but it altogether depended on the extent to which it was destroyed as to the amount of injury or death resulting.

He did not wish to be understood as saying that the person who inhaled this gas had all his blood so vitiated in quality as to cause sufficient poisoning to produce death, but that the structural change was complete so far as it went, and if carried sufficiently far would always cause death. He was sure that many cases of poisoning, of debility, decline and death had resulted from this agent, which he thought the most dangerous of anæsthetics.

Dr. Atkinson said that he was sure of one thing,—that no one would go from here to give nitrous oxide as freely as formerly.

Dr. Odell of New York, thought the differences between the speakers was one of fine points rather than real issues. What was brought out here demonstrated that there was no end to real hard study. When he got his degree of D. D. S., he thought he was a finished man, but he found out that it was easy to take a degree, but very difficult to know any thing. He had found the best plan with his patients, was to persuade them, in proportion as he inspired them with confidence in him, and love for him; in that degree he found it easy to influence them to bear the pain of such operations as were necessary in his practice.

Prof. Taft joined in the protest against anæsthetics. Experiments abundantly proved that the practice of administer ing these was wrong. He knew personally of injury from both nitrous oxide gas and ether. He had known the former to produce hopeless insanity in a few hours. Death occurred in the hands of the best surgeons of the world from chloroform, in spite of all precautions, and in the face of careful examinations for heart and other affections. He cited the case of a patient who had inhaled the gas four years ago, and who said he had never felt like himself since. We judge wrongly that, because death does not take place instantly, therefore no injury is done. The numerous lingering deaths, cases of serious illness, of loss of voice, and other disasters were warnings all should heed. Many of the best operators get along and succeed without the gas, and most of the teeth that are removed there is no necessity for—they could be saved. He had seen pecks of teeth extracted that could have been kept in the mouth. This he thought was one of the most objectionable features of this nefarious practice—the temptation to the wholesale extraction of teeth which ought not to be taken out.

Dr. Evans, of Baltimore, asked Prof. Taft how many times he had used the gas, and was answered, perhaps twenty times.

Dr. Evans said, that he had never given the gas himself, but he certainly was surprised at this wholesale condemnation of and opposition to anæsthetics. The difficulty was not he felt in the majority of cases of death, that the agent was so dangerous a thing, as that the operator was a bungler. He referred particularly to chloroform, and extoled it as one of the grandest medical discoveries, the means of the mitigation and amelioration of more human suffering than all the discoveries of ancient and modern times.

Dr. Coy agreed with Dr. Evans.

Dr. Taft thought one need not give gas at all in order to be a judge of its effects.

Dr. M. S. King, of Pittsburg, always gets mad when he thinks of gas, its troublesomeness and the annoyance and injury of it.

Dr. Templeton, of Pittsburg, stated that he had always made it a rule to give the chloroform himself, deeming himself the equal of the physician. If the patient wanted the physician to give it, it must be done elsewhere than at his office; in that place he was the chief.

Dr. Walker, of New Orleans, objected to nitrous oxide on account of the brevity of the anæsthesia, necessitating hurry and rendering accidents more liable. He had seen, and in this he agreed with Dr. Taft, many cases of injury from the administration of gas, gradual decline and obscure pathological results.

Dr. Evans thought that the cause of much of this trouble with dentists from chloroform, was the result of posture, the upright position being a bad one. He disagreed with the writer of the paper on anæsthetics (Dr. Thompson,) in that he, (Dr. Evans,) considered the pre-administration of whiskey as important, hastening the result, and rendering a less dose efficient.

After some remarks by Dr. Caldwell the meeting adjourned.

---

